# Quantitative research on the impact of COVID‐19 on frontline nursing staff at a military hospital in Saudi Arabia

**DOI:** 10.1002/nop2.1297

**Published:** 2022-07-22

**Authors:** Loujain Sharif, Khalid Almutairi, Khalid Sharif, Alaa Mahsoon, Maram Banakhar, Salwa Albeladi, Yaser Alqahtani, Zalikha Attar, Farida Abdali, Rebecca Wright

**Affiliations:** ^1^ Faculty of Nursing King Abdulaziz University Jeddah Saudi Arabia; ^2^ King Fahad Armed Forces Hospital (KFAFH) Jeddah Saudi Arabia; ^3^ Johns Hopkins School of Nursing Baltimore Maryland USA

**Keywords:** anxiety, COVID‐19, depression, job satisfaction, nurses, Saudi Arabia, stress

## Abstract

**Aim:**

The aim of the study was to examine the relationship between stress, psychological symptoms and job satisfaction among frontline nursing staff at a military hospital in Saudi Arabia during the COVID‐19 pandemic.

**Design:**

Descriptive cross‐sectional study.

**Methods:**

Data were collected using an online survey. All Registered Nurses (*N* = 1,225) working at a military hospital between February to April 2021 were contacted, 625 responded (51%). Data were analysed using descriptive and multivariate analysis, Student's t‐test for independent samples and one‐way analysis of variance followed by Tukey's multiple comparison tests.

**Results:**

Stress was experienced more significantly than depression or anxiety. Approximately 29% of the change in scores for psychological symptoms was explained by age group, being a Saudi national and working in emergency departments (*F*_[3,620]_ = 19.063, *p* < 0.0001). A 37% change in nursing stress scores was explained by nationality and work department. (*F*_[5,618]_ = 19.754, *p* < 0.0001). A 29% change in job satisfaction scores was explained by nationality and work department (*F*_[3,620]_ = 19.063, *p* < 0.0001).

## 1 INTRODUCTION

Saudi Arabia reported its first case of coronavirus disease 2019 (COVID‐19) on March 2, 2020 (Reuters Staff, 2020; Zu et al., 2020). The World Health Organization has identified the COVID‐19 outbreak as a public health emergency and global pandemic (World Health Organization, 2020). The impact of COVID‐19 on those who have contracted it received rapid investigation and documentation (Harper et al., 2020). However, healthcare workers were quickly recognized to be experiencing a secondary impact of COVID‐19, owing to vulnerability to stressors such as inadequate resources, long shifts, sleep problems, work−life imbalances and new occupational hazards (Sasangohar et al., 2020). Notably, previous research on the impact of other coronavirus syndromes (severe acute respiratory syndrome, Middle East respiratory syndrome) found that approximately 62% of healthcare workers reported general health concerns, fear, insomnia, psychological distress, burnout, anxiety, depressive symptoms, posttraumatic stress disorder, psychosomatic symptoms and perceived stigma (Sasangohar et al., 2020).

Compared with other healthcare professionals, nursing staff are particularly susceptible to the negative impact of a pandemic, with a higher vulnerability to negative outcomes associated with working in high‐risk departments (Shaukat et al., 2020). Moreover, the impact is not limited to psychological effects. One systematic review on estimated COVID‐19 infections and deaths among healthcare workers reported 37.2 deaths per 100 infections in nursing staff aged at least 70 years (Bandyopadhyay et al., 2020). Another study conducted in the UK found that out of 157 COVID‐19‐related deaths among medical health workers, 48 (30.6%) were nurses (Kursumovic et al., 2020). This combination of physical (e.g. infection transmission and the underlying manifestations) and psychological effects (e.g. burnout, stress, anxiety and depression) caused by the pandemic (Hu et al., 2020) has led to substantial concerns for nursing staff, with statistically significant bearing on job satisfaction (Del Carmen Giménez‐Espert et al., 2020).

## 2 BACKGROUND

There has been a concerted effort in Saudi Arabia to understand and mitigate the impact of COVID‐19 on nursing staff, with studies investigating stress, fear of infection and resilience in relation to COVID‐19 (Tayyib & Alsolami, 2020); stress and coping strategies in dealing with COVID‐19 (Muharraq, 2021); and nursing knowledge and anxiety related to COVID‐19 (Alsharif, 2021). However, these studies give descriptive statistics with relatively small samples of less than 300 nurses, and, to the best of our knowledge, no study has yet focused on assessing multiple psychological symptoms (depression, anxiety, and stress) collectively in relation to job satisfaction. Furthermore, the effects of COVID‐19 among nursing staff in military hospitals have not yet been explored.

This is a key setting for investigation, as military hospitals in Saudi Arabia are considered highly specialized healthcare organizations, providing all forms of health care to an exclusive population of military personnel and their family members (Walston et al., 2008). Healthcare providers recruited for military hospitals must meet high standards and requirements that differ from those in non‐military care settings (Olenick et al., 2015). Because of higher standards and higher pay levels compared with other healthcare organizations in Saudi Arabia, military hospitals often employ healthcare providers, and nurses in particular, from different countries worldwide (Almalki et al., 2011). Despite the higher salaries and expectations of care associated with urgent needs, military hospitals have had to adapt their policies and protocols in response to greater and new patient needs as a result of COVID‐19. Therefore, these hospitals have also been impacted by the brutal reality, thereby leading to an increase in resignations among nursing staff. Probable reasons for this increase include greater workloads, mandatory overtime, withholding of annual leave and switching of nurses from less demanding areas (e.g. outpatient clinics) to more demanding care areas (e.g. inpatient units), along with the risk of contracting COVID‐19 (King Fahad Armed Forces Hospital, 2020). These changes suggest that nursing staff at military hospitals have experienced many of the same mental and physical side effects as nurses in non‐military hospitals, with the same consequential burnout and resignations. However, it is also commonly reported that nurses avoid seeking psychological support and services (Knaak et al., 2017). This may be due to a fear of stigma and discrimination in the workplace, where needing mental health help can be perceived as weakness (Jones et al., 2020), which is a phenomenon that is particularly common among military personnel (Hernandez et al., 2014).

Despite investigations into the types of symptoms experienced by nursing staff as outlined above, few studies have explored the relationship between psychological impact and nurses' job satisfaction within the context of military hospitals in the Middle East. Therefore, the present study aimed to examine the relationships within and between stress, psychological symptoms (including depression and anxiety) and job satisfaction among frontline nursing staff at a military hospital in Saudi Arabia during the COVID‐19 pandemic. The purpose of this study was to identify key components that may benefit not only the study site in improving nursing staff retention but also the wider healthcare field, as nursing retention is an increasingly documented challenge. We hypothesized that the abovementioned challenges encountered by nurses, as a secondary impact of COVID‐19, are likely to be linked to low job satisfaction among frontline nurses.

## 3 STUDY

### 3.1 Design

We used a descriptive cross‐sectional design with a quantitative questionnaire. Convenience sampling was used to recruit Registered Nurses (RNs) working in all hospital units. Overall, 1,125 RNs worked at the study site. The hospital only has full‐time RNs and does not employ part‐time or agency RNs. As such there was no criteria excluding any RN employed at the hospital from participation in this study. Five hundred seventy‐six participants were required for a 50% response rate (Sataloff & Vontela, 2021). Data were collected from one military healthcare organization in the western region of Saudi Arabia. The hospital provides all medical services with a 420‐bed capacity, serving members of the Saudi Arabian Armed Forces and their families. The hospital is accredited by the Central Board for Accreditation of Healthcare Institutions, Joint Commission International and International Organization for Standardization, and it is the only adult cardiac surgical facility in the western region.

### 3.2 Method

The questionnaire comprised four sections and was in English language, with 122 items, in total and took approximately 35 minutes to complete.

*Section 1 – Demographic information*: We collected data on eight items: age, gender, marital status, nationality, education level, experience and department.

*Section 2 – Expanded Nursing Stress Scale (ENSS;* French et al., 2000): The ENSS (Cronbach's alpha = 0.96) identifies the sources and frequency of stress among hospital nurses. The scale comprises a total of 57 items on the following stressful situations: death and dying patients (7 items), conflict with physicians (5 items), inadequate emotional preparation (3 items), problems related to peers (6 items), problems related to supervisors (7 items), workload (9 items), uncertainty concerning treatment (9 items), patients and their families (8 items) and discrimination (3 items). The ENSS was also used in the present study to assess the frequency in which nurses experienced work stressors, rated within a range between 0–4, on a scale modified from the original as follows: *I have not encountered it* (0), *never stressful* (1), *occasionally stressful* (2), *frequently stressful* (3) and *always stressful* (4). In a pilot test of the modified ENSS, conducted by the authors of this study, the Cronbach's alpha was 0.98.

*Section 3 – Depression*, *Anxiety and Stress Scales (DASS;* Lovibond & Lovibond, 1995): The DASS (Cronbach's alpha = 0.89) focuses on assessing depression, anxiety and stress among hospital nurses. Each of the three scales contains seven items. The depression scale assesses dysphoria, hopelessness, devaluation of life, self‐deprecation, lack of interest/involvement, anhedonia and inertia. The anxiety scale assesses autonomic arousal, skeletal muscle effects, situational anxiety and subjective experience of anxious affect. The stress scale assesses difficulty relaxing, nervous arousal and being easily upset/agitated, irritable/over‐reactive and impatient. The DASS is rated on a scale ranging between 0–3: (0) *does apply to me at all*, (1) *applies to me to some degree or some of the time*, (2) *applies to me to a considerable degree or a good part of time* and (3) *applies to me very much or most of the time*. Cronbach's alpha for the DASS in the current study was calculated as 0.969, indicating excellent reliability.

*Section 4 – Job Satisfaction Survey (JSS;* Spector, 1985): The JSS (Cronbach's alpha 0.91) assesses job satisfaction among hospital nurses. It includes 36 items with nine facets as follows: pay (4 items), promotion, supervision (4 items), fringe benefits (4 items), contingent rewards (4 items), operating procedures (4 items), co‐workers (4 items), nature of work (4 items) and communication (4 items). Items are rated on a six‐point Likert scale with responses ranging from 1 (*disagree very much*) to 6 (*agree very much*). The JSS demonstrated acceptable reliability in the current study, with a Cronbach's alpha of 0.798. Regarding the scoring system, scores for each four‐item subscale ranged from 4 to 24 and were scored as follows: dissatisfied (4–12 points), ambivalent (12–16) and satisfied (16–24). For the total 36‐item JSS, scores ranged from 36 to 216 and were scored as follows: dissatisfied (36–108 points), ambivalent (108–144) and satisfied (144–216; Spector, 1994).

### 3.3 Data collection process

After obtaining ethical approval, potential study participants who were recruited to participate through unit meetings by the head nurses of the units, who acted as gatekeepers. All relevant information on the study, including its research topic, aim, sample and significance were explained to all RNs in each unit. Within Saudi culture, in addition to communication modalities such as email, social media platforms are a common and effective method of communicating with groups within different organizations. Therefore, the head nurse in each unit sent the survey using google form as an electronic link via the social media application “WhatsApp” to all RNs who agreed to participate in the study. The survey was sent out in February 2021 and remained available until April 2021.

### 3.4 Analysis

Data were analysed using SPSS 26.0 Windows version statistical software (IBM, Armonk, NY, USA). Descriptive statistics (means, standard deviations, frequencies and percentages) were used to describe the quantitative and categorical variables. Student's t‐test for independent samples was used to compare the mean values of quantitative outcome variables in relation to the categorical study variable with two categories. One‐way analysis of variance, followed by Tukey's multiple comparison tests (Tukey, 1953), was used to compare the mean values of quantitative outcome variables in relation to the categorical study variables with more than two categories. A *p*‐value of ≤0.05 was used to report the statistical significance of the results.

For the multivariate analysis, a stepwise Multiple linear regression was carried out to observe the independent relationship of variables of categorical study variables with the three quantitative variables (DASS, ENSS and JSS scores). As the study variables were categorical, dummy variables were created to include them in the model. The proportion of variability *R* ^2^ was used to observe the change in the outcome variable explained by the significant independent variables in the model. Regression coefficients were used to observe changes in the outcome variables. A *p*‐value ≤0.05, was used to report the statistical significance of the estimates.

### 3.5 Ethics

Ethical approval was obtained from the King Fahd Armed Forces Hospital‐ Jeddah, Research and Ethics Committee (Ref. number: REC 398), confirming no risk to study participants via the application of an anonymous online survey. The cover page of the survey provided key information, including the importance and purpose, expected time necessary to complete the survey, and why survey recipients were asked to participate. A statement regarding confidentiality and anonymity was included within the online link to the survey. No financial incentives were offered.

## 4 RESULTS

Of the 624 nurses who completed the survey (response rate: 51%), 91.3% were women, approximately two‐thirds (66.8%) were aged between 25–35 years, and more than 50% were unmarried. The majority were Filipino (75.8%), and only 5.6% were Saudi. Approximately 90% of the sample had a bachelor's degree, and 48.4% had 1–5 years of experience; 6.3% had more than 15 years of experience. The sample was distributed among the following departments and units: emergency departments (14.6%), intensive care units (22.6%), inpatient units (39.1%) and outpatient units (9.6%); the remaining 14.1% were from other departments. A quarter of the sample (n = 156) had tested positive for COVID‐19 (Table 1).

**TABLE 1:** Socio‐demographic and professional characteristics of participants (*N* = 624)

Characteristics	*N* (%)
Age groups	
25–30	198 (31.7)
31–35	219 (35.1)
36–40	79 (12.7)
41–45	56 (9.0)
46–50	49 (7.9)
≥51	23 (3.7)
Gender	
Male	54 (8.7)
Female	570 (91.3)
Marital status	
Single	326 (52.2)
Married	273 (43.8)
Separated/Divorced	25 (4.0)
Nationality	
Filipino	473 (75.8)
Indian	87 (13.9)
Malaysian	11 (1.8)
Saudi	35 (5.6)
South African	18 (2.9)
Education level	
Diploma	46 (7.4)
Bachelor	564 (90.4)
Master	14 (2.2)
Number of years of experience	
<1	6 (1.0)
1–5	302 (48.4)
6–10	180 (28.8)
11–15	97 (15.5)
>15	39 (6.3)
Place of working	
Emergency department	91 (14.6)
Intensive care units	141 (22.6)
Outpatient units	60 (9.6)
Inpatient units	244 (39.1)
Others	88 (14.1)
Have tested positive for COVID‐19	
Yes	156 (25.0)
No	478 (75.0)

Table 2 shows the mean values of the three DASS subscales (depression, anxiety and stress). The mean stress score was higher than the mean scores for either depression or anxiety. Table 3 shows the ENSS scores and mean values of its nine domains, in which the mean score of the “workload” domain was highest (2.39), followed by mean scores of “patients and their families” (2.30) and “problems relating to supervisors” (2.14); the mean scores of the remaining six domains were less than 2.0 The mean value for the nine domains of the JSS was 121.07 (22.1), which indicated ambivalence (Table 4). The only mean score that indicted satisfaction was in the “nature of work” domain (17.04), followed by “co‐workers” (15.88) and “supervision” (15.16). The mean scores of the remaining six domains were less than 15.0, ranging from ambivalent to dissatisfied.

**TABLE 2:** Comparison of mean scores of DASS sub scales and total score in relation to socio‐demographic and professional characteristics of study subjects (*n* = 624)

Characteristics	DASS sub scales						Total score	
	Depression		Anxiety		Stress			
	9.92 (9.8)^a^		9.81 (10.3)^a^		11.31 (10.2)^a^			
	Mean (SD)	*p*‐value	Mean (SD)	*p*‐value	Mean (SD)	*p*‐value	Mean (SD)	*p*‐value
Age groups								
25–30	11.1 (10.4)	**0.03**	11.6 (10.2)	**0.018**	12.5 (1.4)	**0.035**	35.2 (30.1)	**0.027**
31–35	10.1 (10.9)		9.7 (9.8)		11.1 (10.9)		30.1 (30.5)	
36–40	9.9 (9.8)		10.0 (9.5)		11.6 (10.4)		31.5 (28.9)	
41–45	7.4 (9.5)		8.3 (9.4)		10.0 (9.3)		25.7 (27.2)	
46–50	6.3 (5.9)		6.7 (6.5)		7.3 (6.3)		20.2 (17.4)	
≥51	9.0 (10.7)		7.9 (11.1)		12.9 (9.9)		29.8 (31.0)	
Gender								
Male	9.96 (9.5)	0.908	9.8 (9.2)	0.913	10.7 (9.9)	0.652	30.4 (27.9)	0.878
Female	9.8 (10.3)		9.9 (9.8)		11.4 (10.3)		31.1 (29.4)	
Marital status								
Single	9.8 (10.2)	0.954	9.8 (9.5)	0.856	11.1 (10.2)	0.761	30.7 (28.9)	0.953
Married	9.8 (10.4)		10.1 (10)		11.5 (10.4)		31.4 (29.9)	
Separated/Divorced	9.2 (9.3)		9.3 (9.6)		12.4 (9.8)		30.9 (27.4)	
Nationality								
Filipino	9.4 (10.1)	**0.009**	9.3 (9.4)	**<0.001**	10.7 (10.1)	**0.001**	29.4 (28.6)	**0.001**
Indian	10.3 (8.6)		10.9 (8.2)		12.0 (8.3)		33.2 (24.2)	
Malaysian	13.1 (13.1)		14.9 (13.8)		14.5 (12.8)		42.5 (39.5)	
Saudi	15.2 (14.1)		15.9 (13.8)		17.8 (13.8)		48.9 (40.7)	
South African	6.8 (8.2)		5.3 (7.7)		9.1 (10.0)		21.2 (25.3)	
Education level								
Diploma	8.1 (8.3)	0.494	6.8 (8.4)	0.057	10.4 (9.1)	0.643	25.3 (24.8)	0.381
Bachelor	9.9 (10.4)		10.2 (9.8)		11.3 (10.4)		31.5 (29.7)	
Master	9.6 (10.1)		8.1 (7.9)		13.3 (10.2)		31.0 (26.4)	
Number of years of experience								
< 1	8.0 (10.6)	**0.045**	10.3 (8.3)	**0.049**	9 (7.1)	0.34	27.3 (24.5)	0.095
01‐May	9.6 (9.8)		10 (9.5)		11.2 (9.9)		30.7 (28.2)	
06‐Oct	11.4 (11.5)		10.9 (10.4)		12.3 (11.6)		34.5 (32.6)	
Nov‐15	7.5 (8.0)		7.3 (7.8)		9.7 (8.3)		24.5 (22.7)	
>15	10.5 (11.8)		11.3 (12.0)		11.9 (11.2)		33.7 (34.3)	
Place of working								
Emergency department	12.5 (11.5)	**0.001**	12.2 (10.9)	**0.001**	13.8 (11.3)	0.007	38.5 (32.9)	**0.001**
Intensive care units	8.8 (9.5)		9.6 (8.8)		10.8 (9.0)		29.2 (26.3)	
Outpatient units	8.2 (7.6)		7.3 (6.8)		9.5 (8.2)		25.1 (21.0)	
Inpatient units	8.8 (9.5)		8.9 (9.1)		10.3 (10.1)		28.0 (27.7)	
Others	12.6 (12.5)		12.6 (12.2)		13.5 (12.2)		38.7 (35.7)	
Have tested positive for COVID‐19								
Yes	10.5 (9.8)	0.359	11.4 (9.8)	**0.03**	12.2 (10.0)	0.203	34.1 (28.6)	0.136
No	9.6 (10.4)		9.4 (9.7)		11.0 (10.4)		30.0 (29.5)	

*Note*: Bolded text denotes *p* value of <0.05.

^a^ Depression, Anxiety, and Stress scale (DASS) subscale mean scores and standard deviations (SD).

**TABLE 3:** Comparison of mean values of nine domains and total score of ENSS scale in relation to socio‐demographic and professional characteristics of study subjects (*n* = 624)

Characteristics	Death and dying		Conflicts with physicians		Inadequate emotional preparation		Problems relating to peers		Problems relating to supervisors		Workload		Uncertainty concerning treatment		Patients and their families		Discrimination		Total ENSS score	
	1.63 (1.0)^a^		1.93 (1.0)^a^		1.86 (0.95)^a^		1.74 (0.97)^a^		2.14 (1.17)^a^		2.39 (0.99)^a^		1.97 (1.01)^a^		2.30 (1.08)^a^		1.42 (1.10)^a^		17.40 (8.22)^a^	
	Mean (SD)	*p*‐value	Mean (SD)	*p*‐value	Mean (SD)	*p*‐value	Mean (SD)	*p*‐value	Mean (SD)	*p*‐value	Mean (SD)	*p*‐value	Mean (SD)	*p*‐value	Mean (SD)	*p*‐value	Mean (SD)	*p*‐value	Mean (SD)	*p*‐value
Age groups																				
25–30	1.6 (1.0)	0.473	1.9 (1.0\)	0.826	1.9 (1.0)	0.52	1.8 (1.0)	0.609	2.1 (1.2)	0.89	2.4 (1.1)	0.744	1.9 (1.1)	0.917	2.3 (1.2)	0.945	1.5 (1.2)	0.144	17.4 (8.9)	0.839
31–35	1.6 (0.9)		1.9 (0.9)		1.8 (0.8)		1.7 (0.9)		2.2 (1.1)		2.3 (0.9)		1.9 (0.9)		2.3 (1.0)		1.3 (1.0)		17.2 (7.7)	
36–40	1.7 (1.0)		2.1 (1.0)		1.9 (1.0)		1.7 (0.9)		2.2 (1.2)		2.4 (0.9)		2.1 (1.0)		2.4 (1.0)		1.4 (1.1)		17.9 (8.2)	
41–45	1.5 (1.0)		1.9 (0.9)		1.8 (0.9)		1.6 (0.9)		2.1 (1.1)		2.4 (0.9)		1.9 (0.9)		2.3 (1.1)		1.2 (1.0)		16.7 (7.9)	
46–50	1.7 (1.0)		1.8 (1.1)		1.7 (0.9)		1.7 (1.0)		2.1 (1.2)		2.3 (0.9)		1.9 (0.9)		2.3 (1.1)		1.6 (1.0)		17.3 (8.4)	
> = 51	1.9 (1.0)		1.9 (0.9)		2.2 (1.0)		1.9 (0.9)		2.3 (1.0)		2.7 (0.7)		2.0 (0.9)		2.4 (0.9)		1.7 (1.1)		19.2 (7.8)	
Gender																				
Male	1.7 (0.9)	0.378	2.0 (1.0)	0.567	1.9 (0.9)	0.945	1.7 (0.9)	0.828	2.2 (1.2)	0.815	2.4 (1.0)	0.822	2.0 (1.0)	0.749	2.4 (1.1)	0.34	1.5 (1.1)	0.363	17.9 (8.0)	0.65
Female	1.6 (1.0)		1.9 (1.0)		1.8 (0.9)		1.7 (0.9)		2.1 (1.1)		2.3 (1.0)		1.9 (1.0)		2.3 (1.1)		1.4 (1.1)		17.3 (8.2)	
Marital status																				
Single	1.5 (0.9)	0.031	1.9 (0.9)	0.322	1.9 (0.9)	0.558	1.7 (0.9)	0.824	2.1 (1.1)	0.935	2.4 (0.9)	0.547	2.0 (0.9)	0.836	2.3 (1.0)	0.616	1.4 (1.1)	0.216	17.4 (7.9)	0.549
Married	1.7 (1.0)		1.8 (1.0)		1.8 (1.0)		1.7 (1.0)		2.1 (1.2)		2.3 (1.0)		1.9 (1.0)		2.3 (1.1)		1.4 (1.1)		17.2 (8.6)	
Separated/Divorced	2.0 (1.1)		2.1 (0.9)		2.1 (1.1)		1.8 (1.0)		2.2 (1.2)		2.5 (0.9)		2.0 (1.0)		2.4 (0.9)		1.8 (1.3)		19.1 (8.5)	
Nationality																				
Filipino	1.7 (1.0)	**0.001**	2.0 (0.9)	**0.001**	1.9 (0.9)	**0.001**	1.8 (0.9)	**0.001**	2.3 (1.1)	**0.001**	2.5 (0.9)	**0.001**	2.1 (0.9)	**0.001**	2.5 (1.0)	**0.001**	1.5 (1.1)	**0.001**	18.4 (8.0)	0.001
Indian	1.3 (0.8)		1.3 (0.8)		1.4 (0.9)		1.3 (0.7)		1.2 (0.9)		1.6 (0.8)		1.2 (0.8)		1.5 (0.8)		0.9 (0.8)		11.9 (6.8)	
Malaysian	1.9 (1.1)		2.4 (0.9)		2.2 (0.9)		2.2 (0.9)		2.6 (1.0)		2.6 (0.9)		2.2 (0.9)		2.4 (1.0)		1.7 (0.5)		20.4 (7.2)	
Saudi	2.2 (1.1)		2.0 (1.1)		2.0 (1.1)		1.8 (1.2)		1.9 (1.1)		2.3 (1.0)		2.1 (1.1)		1.8 (1.1)		1.4 (1.2)		17.9 (9.0)	
South African	1.0 (0.7)		1.6 (0.9)		1.8 (1.3)		1.5 (1.0)		1.7 (1.4)		2.2 (1.2)		1.4 (0.9)		2.3 (1.1)		0.8 (0.6)		13.9 (8.1)	
Education level																				
Diploma	1.6 (1.1)	0.952	1.7 (1.1)	0.328	1.9 (1.0)	0.731	1.6 (1.1)	0.807	1.9 (1.3)	0.117	2.2 (1.0)	0.203	1.7 (1.1)	0.14	2.0 (0.9)	0.135	1.2 (1.0)	0.304	15.9 (8.7)	0.374
Bachelor	1.6 (1.0)		1.9 (0.9)		1.8 (0.9)		1.7 (0.9)		2.2 (1.2)		2.4 (0.9)		2.0 (1.0)		2.3 (1.1)		1.4 (1.1)		17.5 (8.1)	
Master	1.5 (1.2)		1.8 (1.2)		1.7 (1.3)		1.7 (1.3)		1.7 (1.2)		2.1 (1.2)		1.8 (1.3)		2.2 (1.2)		1.6 (1.4)		16.1 (10.5)	
Number of years of experience																				
< 1	1.2 (1.1)	**0.001**	1.1 (1.3)	**0.009**	1.5 (1.3)	0.287	1.8 (1.2)	**0.039**	1.4 (1.3)	0.013	1.6 (1.2)	**0.041**	1.6 (1.2)	0.112	1.3 (1.1)	**0.031**	1.3 (1.4)	0.121	12.9 (10.4)	**0.019**
01‐May	1.5 (0.9)		1.8 (0.9)		1.8 (1.0)		1.6 (0.9)		2.0 (1.2)		2.3 (1.0)		1.9 (1.0)		2.2 (1.1)		1.3 (1.1)		16.4 (8.4)	
06‐Oct	1.8 (1.0)		2.1 (0.9)		19 (0.8)		1.9 (0.9)		2.3 (1.1)		2.5 (0.9)		2.1 (0.9)		2.4 (1.0)		1.5 (1.0)		18.6 (7.7)	
Nov‐15	1.7 (1.0)		1.9 (1.0)		1.8 (0.9)		1.7 (0.9)		2.3 (1.1)		2.4 (0.9)		2.0 (0.9)		2.4 (1.0)		1.5 (1.0)		17.8 (8.1)	
>15	2.0 (1.0)		2.1 (1.0)		2.0 (1.0)		1.9 (1.0)		2.1 (1.1)		2.5 (1.0)		2.1 (1.1)		2.4 (1.0)		1.6 (1.2)		18.8 (8.7)	
Place of working																				
Emergency department	2.1 (0.8)	**0.001**	2.4 (0.9)	**0.001**	2.2 (0.9)	**0.001**	2.2 (0.9)	**0.001**	2.8 (1.0)	**0.001**	2.9 (0.8)	**0.001**	2.4 (0.9)	**0.001**	2.9 (0.9)	**0.001**	1.8 (1.0)	**0.001**	21.7 (7.5)	**0.001**
Intensive care units	1.6 (0.9)		1.8 (0.9)		1.7 (0.8)		1.6 (0.8)		2.0 (1.1)		2.1 (0.9)		1.7 (0.9)		1.9 (1.0)		1.2 (1.0)		15.9 (7.5)	
Outpatient units	1.4 (1.0)		1.7 (0.8)		1.9 (0.8)		1.7 (0.8)		2.2 (1.0)		2.4 (0.9)		1.8 (0.9)		2.6 (0.8)		1.7 (1.1)		17.4 (6.6)	
Inpatient units	1.4 (1.0)		1.8 (1.0)		1.7 (1.0)		1.5 (0.9)		1.9 (1.2)		2.2 (1.0)		1.9 (1.0)		2.1 (1.1)		1.2 (1.1)		15.8 (8.1)	
Have tested positive for COVID‐19																				
Yes	1.7 (0.9)	0.078	2.0 (0.9)	0.181	1.9 (0.9)	0.704	1.8 (0.9)	0.13	2.2 (1.2)	0.526	2.4 (0.9)	0.373	2.0 (0.9)	0.368	2.4 (1.0)	0.145	1.5 (1.0)	0.111	18.1 (7.9)	0.188
No	1.6 (1.0)		1.9 (1.0)		1.8 (0.9)		1.7 (0.9)		2.1 (1.2)		2.3 (1.2)		1.9 (1.0)		2.3 (1.1)		1.4 (1.1)		17.1 (8.3)	

*Note*: Bolded text denotes *p* value of <0.05.

^a^ Extended Nursing Stress Scale (ENSS) mean scores and standard deviations (SD),

**TABLE 4:** Comparison of mean values of nine domains and total score of job satisfaction scale in relation to socio‐demographic and professional characteristics of study subjects (*n* = 624)

Characteristics	Pay		Promotion		Supervision		Fringe benefits		Contingent rewards		Operating conditions		Co‐workers		Nature of work		Communication		Total score	
	12.59 (3.9)^a^		12.44 (3.5)^a^		15.16 (4.8)^a^		10.98 (3.9)^a^		11.67 (3.8)^a^		11.10 (2.6)^a^		15.88 (3.7)^a^		17.04 (3.8)^a^		14.19 (4.2)^a^		121.07 (22.1)^a^	
	Mean (SD)	*p*‐value	Mean (SD)	*p*‐value	Mean (SD)	*p*‐value	Mean (SD)	*p*‐value	Mean (SD)	*p*‐value	Mean (SD)	*p*‐value	Mean (SD)	*p*‐value	Mean (SD)	*p*‐value	Mean (SD)	*p*‐value	Mean (SD)	*p*‐value
Age groups																				
25–30	12.4 (3.8)	0.468	12.7 (3.4)	0.173	15.1 (4.7)	0.578	10.9 (3.7)	0.595	11.8 (3.7)	0.968	10.9 (2.8)	0.433	15.5 (4.1)	0.177	15.6 (3.8)	**<0.001**	14.0 (4.2)	0.759	119.2 (22.2)	0.498
31–35	12.4 (4.2)		12.5 (3.6)		15.2 (5.0)		10.7 (4.2)		11.5 (4.0)		11.0 (2.5)		16.0 (3.6)		17.0 (3.7)		14.2 (4.2)		120.8 (22.7)	
36–40	12.7 (3.8)		12.5 (3.2)		14.6 (5.0)		11.7 (3.7)		11.7 (3.9)		11.5 (2.7)		15.5 (3.7)		16.9 (3.9)		14.2 (4.0)		121.2 (22.2)	
41–45	13.5 (3.7)		11.6 (3.7)		16.1 (4.2)		10.8 (3.1)		11.9 (3.6)		11.0 (2.3)		16.6 (3.2)		18.9 (3.3)		15.0 (4.4)		125.4 (19.3)	
46–50	12.6 (3.9)		12.6 (3.5)		14.8 (5.4)		11.1 (4.0)		11.5 (4.2)		11.3 (2.2)		16.7 (3.2)		18.8 (3.3)		14.0 (4.4)		123.5 (22.9)	
≥51	13.3 (4.3)		11.1 (3.1)		14.9 (4.5)		11.2 (3.4)		11.8 (3.0)		11.7 (2.9)		16.0 (2.9)		19.0 (3.7)		14.1 (4.4)		123.2 (18.6)	
Gender																				
Male	12.1 (4.8)	0.35	12.2 (3.8)	0.568	13.3 (6.0)	**0.004**	10.3 (4.0)	0.215	10.8 (3.9)	0.096	11.6 (2.9)	0.18	15.5 (4.0)	0.449	16.4 (4.5)	0.181	13.2 (4.7)	0.077	115.5 (27.2)	0.053
Female	12.6 (3.9)		12.5 (3.4)		15.3 (4.7)		11.0 (3.9)		11.7 (3.8)		11.1 (2.5)		15.9 (3.7)		17.1 (3.8)		14.3 (4.1)		121.6 (21.4)	
Marital status																				
Single	12.3 (3.8)	0.183	12.3 (3.4)	0.568	15.1 (4.8)	0.556	10.8 (3.9)	0.443	11.6 (3.8)	0.46	11.0 (2.5)	0.193	15.9 (4.1)	0.994	16.8 (3.9)	**0.005**	14.2 (4.2)	0.564	119.9 (22.9)	0.304
Married	12.8 (4.2)		12.6 (3.6)		15.2 (5.0)		11.2 (3.8)		11.8 (3.8)		11.2 (2.7)		15.9 (3.3)		17.1 (3.6)		14.1 (4.2)		121.9 (21.1)	
Separated/Divorced	13.3 (4.4)		12.2 (3.0)		16.2 (4.8)		11.2 (3.9)		10.9 (3.0)		11.9 (2.2)		15.8 (2.6)		19.3 (3.9)		15.0 (3.9)		125.7 (20.0)	
Nationality																				
Filipino	12.1 (4.0)	**<0.001**	12.0 (3.3)	**<0.001**	14.4 (4.9)	**<0.001**	10.4 (3.8)	**<0.001**	11.4 (3.8)	**0.007**	11.0 (2.5)	**0.03**	16.1 (3.7)	**0.005**	17.2 (3.9)	**0.002**	13.9 (4.3)	**0.026**	118.6 (22.9)	**<0.001**
Indian	14.3 (3.4)		14.4 (3.5)		17.6 (3.4)		13.5 (3.0)		12.7 (3.4)		11.0 (2.8)		15.5 (3.5)		16.0 (3.4)		15.4 (3.5)		130.6 (15.9)	
Malaysian	13.2 (2.6)		12.9 (2.0)		16.2 (3.2)		13.2 (1.5)		13.4 (3.2)		13.2 (2.9)		12.2 (3.1)		15.1 (2.4)		13.5 (2.9)		122.8 (14.3)	
Saudi	13.9 (3.3)		14.0 (2.4)		16.4 (4.1)		12.0 (3.5)		12.5 (3.4)		11.8 (3.0)		15.2 (3.2)		16.9 (3.7)		14.0 (4.1)		126.8 (16.2)	
South African	13.4 (4.8)		10.4 (4.6)		19.2 (5.3)		10.9 (4.6)		11.8 (4.0)		11.4 (2.7)		15.9 (3.9)		19.5 (4.3)		15.4 (4.5)		128.2 (25.3)	
Education level																				
Diploma	14.5 (4.5)	**0.004**	12.8 (4.0)	0.283	17.3 (4.5)	**0.004**	12.1 (4.3)	0.128	13.1 (3.8)	**0.015**	11.8 (2.9)	0.145	15.6 (3.5)	0.656	17.9 (4.0)	0.229	14.9 (4.2)	0.526	130.1 (20.8)	**0.013**
Bachelor	12.4 (3.9)		12.4 (3.4)		14.9 (4.8)		10.9 (3.8)		11.6 (3.8)		11.0 (2.6)		15.9 (3.7)		16.9 (3.8)		14.1 (4.2)		120.3 (21.9)	
Master	12.6 (4.2)		13.7 (4.0)		16.8 (6.4)		10.4 (4.0)		10.4 (3.1)		11.0 (2.1)		16.8 (3.9)		17.8 (5.7)		14.1 (5.8)		123.6 (28.6)	
Number of years of experience <1																				
01‐May	13.0 (1.8)	0.216	14.3 (1.4)	0.379	16.0 (4.0)	0.701	14.5 (2.8)	0.146	12.5 (4.5)	0.567	11.8 (2.4)	0.376	14.5 (4.5)	0.165	19.2 (4.6)	<0.001	14.2 (1.7)	0.98	129.8 (17.6)	0.677
06‐Oct	12.3 (4.1)		12.6 (3.7)		15.4 (4.8)		10.9 (3.8)		11.7 (3.7)		10.9 (2.6)		16.1 (3.7)		16.6 (3.9)		14.3 (4.3)		120.8 (22.5)	
Nov‐15	12.8 (3.9)		12.1 (3.2)		15.1 (4.9)		10.7 (3.9)		11.6 (3.7)		11.3 (2.7)		15.3 (3.8)		16.8 (3.7)		14.0 (4.1)		119.9 (22.0)	
>15	13.3 (3.6)		12.3 (3.2)		14.6 (5.0)		11.3 (3.6)		11.8 (4.2)		11.2 (2.2)		16.1 (3.2)		18.0 (3.4)		14.2 (4.4)		122.9 (20.9)	
	12.5 (4.0)		12.5 (3.1)		15.0 (4.9)		11.0 (4.3)		10.7 (3.2)		11.5 (2.9)		16.0 (4.2)		18.8 (4.0)		14.4 (4.4)		122.6 (22.8)	
Place of working																				
Emergency department	11.4 (3.9)	0.001	11.5 (3.0)	0.055	11.9 (4.6)	**<0.001**	9.9 (3.6)	**0.009**	9.6 (3.5)	**<0.001**	10.6 (1.8)	**0.001**	14.9 (3.6)	**<0.001**	16.1 (3.8)	0.054	12.6 (3.4)	**<0.001**	108.5 (20.9)	**<0.001**
Intensive care units	12.8 (3.6)		12.7 (3.2)		15.1 (4.2)		11.6 (3.6)		11.7 (3.3)		10.9 (2.3)		15.4 (3.2)		16.8 (3.6)		14.6 (3.8)		121.6 (17.8)	
Outpatient units	11.9 (4.0)		12.2 (3.3)		14.0 (4.6)		10.3 (3.6)		11.2 (3.4)		12.3 (3.0)		17.2 (2.9)		16.8 (3.5)		14.0 (3.8)		119.9 (19.5)	
Inpatient units	13.2 (4.1)		12.6 (3.7)		16.8 (4.7)		11.1 (4.0)		12.6 (3.8)		11.0 (2.6)		16.6 (4.0)		17.4 (3.9)		14.9 (4.5)		126.3 (24.1)	
Others	12.3 (3.8)		12.8 (3.7)		14.8 (4.7)		11.1 (4.2)		11.5 (4.1)		11.3 (3.0)		14.7 (3.5)		17.4 (4.0)		13.5 (4.6)		119.4 (19.9)	
Have tested positive for COVID‐19																				
Yes	12.6 (3.9)	0.83	12.6 (3.7)	0.446	15.2 (5.2)	0.827	11.2 (3.6)	0.453	11.5 (3.7)	0.651	10.9 (2.4)	0.188	15.2 (3.7)	**0.006**	16.6 (3.9)	0.078	13.8 (3.8)	0.109	119.6 (22.1)	0.334
No	12.6 (4.0)		12.4 (3.4)		15.1 (4.7)		10.9 (4.0)		11.7 (3.8)		11.2 (2.6)		16.1 (3.7)		17.2 (3.8)		14.3 (4.3)		121.6 (22.0)	

*Note*: Bolded text denotes *p* value of <0.05.

### 4.1 Bivariate and multivariate analyses

For mean DASS scores, bivariate analysis showed statistically significant differences in relation to age group, nationality and work department with further statistically significant differences found in mean anxiety scores among nurses who had tested positive for COVID‐19 (*p* = 0.030; Table 2). Multivariate analysis revealed that the overall regression model was statistically significant (*F*_[3,620]_ = 19.063, *p* < 0.0001), with an *R* ^2^ of 29.1 (Table S1). The *R* ^2^ is the proportion of variability, which means approximately 29% of the change in DASS scores was explained by age group (25–30 years), being a Saudi national and working in emergency or “other” departments. The corresponding regression coefficients of these variables indicated that the DASS scores increased on average (i) by 6.334 units in nurses aged 20–30 years when compared to those aged 46–50 years, (ii) by 17.725 units in Saudi nationals when compared to South African nationals and (iii) by 11.699 units in nurses who worked in emergency departments when compared to those who worked in outpatient departments (Table S1).

For ENSS scores, bivariate analysis showed statistically significant differences related to nationality, place of work and experience (Table 3). Multivariate analysis showed that the overall regression model was statistically significant (*F*_[5,618]_ = 19.754, *p* < 0.0001) with an *R* ^2^ of 37.1 (Table S2). A 37% change in ENSS score was explained by nationality and place of work. The corresponding regression coefficients of these variables indicated that ENSS scores increased, on average, (i) by 5.619 units in Filipino nationals when compared to Indian nationals, (ii) by 7.987 units in Malaysian nationals when compared to Indian nationals, (iii) by 4.976 units in Saudi nationals when compared to Indian nationals and (iv) by 4.996 units in nurses who worked in emergency departments when compared to those who worked in inpatient departments (Table S2).

For JSS scores, bivariate analysis showed that the mean values had statistically significant differences in relation to nationality, place of work and education level (Table 4). Multivariate analysis showed that the overall regression model was statistically significant (*F*_[3,620]_ = 19.063, *p* < 0.0001), with an *R* ^2^ of 29 (Table S3). A 29% change in JSS score was explained by nationality and place of work. The corresponding regression coefficients of these variables indicated that JSS scores increased, on average, (i) by 13.022 units in Indian nationals when compared with Filipino nationals, (ii) by 10.017 units in Saudi nationals when compared to Filipino nationals and (iii) by 9.992 units in nurses who worked in inpatient departments when compared to those who worked in outpatient departments (Table S3).

## 5 DISCUSSION

The present study explored the impact of COVID‐19 on nurses working in a military hospital in Saudi Arabia and identified correlations between psychological symptoms and job satisfaction. The data give a detailed understanding of specific challenges to enable the study site to give additional support where needed, as well as give the wider field with new insights that can be built upon in future research. We found that the COVID‐19 pandemic is driving frontline nursing staff in the Jeddah region of Saudi Arabia to experience severe psychological strain.

Based on mean DASS scores, stress was the highest, when compared to depression and anxiety. This result is consistent with a meta‐analysis of 93 studies in which stress was found to be the most severe psychological symptom among nurses working during the COVID‐19 pandemic (Al Maqbali et al., 2021). This result itself is unsurprising, as stress is considered a normal reaction to circumstances related to the pandemic, whereas depression and anxiety are considered psychiatric disorders that should meet certain symptom criteria for a specific duration (Regier et al., 2013). However, nurses in the present study, who tested positive for COVID‐19 showed symptoms of anxiety. A previous qualitative exploration with nurses who had contracted COVID‐19 revealed similar results, while also providing further context regarding the depth of anxiety, fear and psychological shock they experienced (He et al., 2021). However, as that was the only qualitative study, we were able to identify on this topic to date, we highlight this as an area that would benefit from further qualitative research not only to determine lived experiences but also to identify mitigating and supporting factors.

Data collected using the ENSS and JSS indicated that the most significant sources of stress for nursing staff in the present study were those associated with their work environment, such as workload, working under pressure, short time allotted to complete tasks, unsuitable rest/work regimens, frequent night shifts and overtime work. Pre‐pandemic, unusually high workloads were countered by reductions in outpatient appointments and treatments. However, the uniquely intense and demanding nature of COVID‐19 has made that an impossibility for isolation and triage hospitals. Similar findings have been reported elsewhere, as continuous emergency COVID‐19 cases, along with sustained increases in the number of suspected and confirmed cases, are placing frontline nursing staff under intense pressure (Brahmi et al., 2020; Kakar et al., 2021). Moreover, the extreme nature of COVID‐19 cases and high mortality rates have also changed the challenges nurses face in their work environment. New infection control safety policies have physically separated patients and families to reduce the risk of cross‐infection (Hsu et al., 2020; Jaswaney et al., 2022). Nurses implementing these policies have at times faced unreasonable demands and even abuse from distressed families, which exacerbates stressors and increases the pressure on them (Abu‐Snieneh, 2021). We found this to be the case among our nursing participants, who reported distress at the manner and frequency of patients deteriorating and dying, regardless of all medical and nursing efforts and care. These encounters led to a sense that the pandemic cannot be overcome, causing some nurses to experience guilt and self‐blame. This phenomenon has been noted elsewhere, as nurses have responded to blaming themselves, distressed, or angry relatives and patients and cited as one of the main stressors among frontline nurses (Byrne et al., 2021; Liu et al., 2020). We suggest that training in end‐of‐life care processes and approaches may be beneficial to give nurses with the skills to care for patients and families and to equip them with resiliency skills for this type of care (Peters et al., 2013).

Frontline nurses were further impacted by the department in which they worked. We found nurses who worked in emergency departments scored the highest on the DASS, and ENSS, which is consistent with another study showing that nurses working in high‐exposure units with suspected COVID‐19 patients had higher levels of depression than nurses working in other units (Doo et al., 2021). There could be several reasons for this finding, such as an unsafe work environment, insufficient personal protective equipment and unknown patient conditions. In addition, emergency departments are known to be unpredictable work environments, which not only means nurses must be ready to respond to any potential patient need but also increases their vulnerability to unexpected events, such as workplace violence and crises (Cui et al., 2021).

There were other multiple domains on the ENSS and JSS that contributed to frontline nurses experiencing occupational stress and lacking job satisfaction, respectively. Interestingly, one correlation that was found was between the level of satisfaction and the level of education. Other researchers have found that the higher the level of education, the higher the level of satisfaction (Coomber & Barriball, 2007). Conversely in the present study, we found that the higher the level of education, the lower the level of satisfaction. One possible explanation for this could be that during the COVID‐19 pandemic, nurses with higher levels of education are more prepared and equipped to understand evidence‐based practice and policies and guidelines, and the absence of such may have contributed towards feelings of distress and lower satisfaction than nurses who are less highly trained and may not be as aware of the lack of research underpinning rapidly developed new policies and guidelines. This finding is at odds with other studies exploring this relationship (Lorber & Skela Savič, 2012). Another possible reason is that “job satisfaction” has not been consistently defined across studies (Coomber & Barriball, 2007), and those previous studies were performed in other counties where the term's meaning may have different cultural nuances.

Another area of note was as a perceived lack of support from supervisors. Although they are generally more experienced than their subordinates, nursing supervisors have been asked to serve in their roles with greater demands on them to manage an unfamiliar scenario (Alnazly et al., 2021). As such, previously developed regulations, protocols and processes have not been effective or appropriate for responding to changing patient needs or care practices for infection control management; thus, supervisors have simply not had the information needed to guide practice and support junior staff, patients and families (Buheji & Buhaid, 2020). We found the nature of relationships to be a consistent source of stress for nurses, with conflicts between co‐workers (nurse to nurse) and with physicians, and a sense of continuous blame directed at nurses being particularly challenging. This is not an unsubstantiated perception, as Wang et al. (2020) found that other medical professionals often treat nurses as scapegoats.

Age was of particular significance in the present study, as depression, anxiety and stress were significantly higher in nurses aged 25–30 years. This is in line with the results of other studies with nurses in Saudi Arabia (Abu‐Snieneh, 2021; Ghawadra et al., 2019) and internationally. For example, in China, Portugal and Turkey, younger frontline nurses were found to be more likely to experience depression and worry about personal or family health during the COVID‐19 pandemic (Murat et al., 2021; Sampaio et al., 2021; Zheng et al., 2021). Potential explanations include a lack of preparedness for the occupational role in a pandemic and less experience responding to crisis situations among younger nurses, compared with older nurses (Shahrour & Dardas, 2020). Within our setting, another possible explanation connects to a prevailing cultural expectation. In Arab cultures it is expected that by age 25, most people will have settled down and established a family. Thus, attempts to meet expectations, such as finding the right partner, during the pandemic while experiencing mental and physical distress is likely to increase the negative psychological impact on individuals in this age group.

Nationality was of particular interest, as although the five nationalities of nurses captured in the questionnaire (Filipino, Indian, Malaysian, Saudi and South African) were not normally distributed, Saudi nurses showed higher levels of depression, anxiety and stress than nurses of other nationalities. Similar findings were reported by Al‐Dossary et al. (2020), whose study on the effect of COVID‐19 in 500 nurses found that non‐Saudi nurses had higher self‐reported awareness, positive attitudes, optimal prevention and positive perceptions compared with Saudi nurses. A possible explanation is that many non‐Saudi nurses working in the region are away from their families, while Saudi nurses are in their usual living arrangements. Therefore, during the pandemic, Saudi nurses have an additional concern of transmitting the virus to their families, while non‐Saudi nationals may be concerned about their loved ones, but do not experience the distress of their job leading to direct risk or harm to them (Abu‐Snieneh, 2021). Other studies have also shown family safety to be a significant concern among frontline nursing staff during the COVID‐19 pandemic (Labrague, 2021).

### 5.1 Limitations

The present study has some limitations that should be noted. Although this study provides insights into the main psychological stressors that are impacting the nursing workforce and to what degree, it would have been strengthened by including a qualitative arm to provide context and depth to our findings. This research is planned as our next phase. Survey tools were delivered in their original English language as our hospital nursing staff includes a wide range of nationalities and English is the official language of Saudi healthcare organizations. However, it may be beneficial in future research to develop alternative translations and variables that would more directly capture cultural context.

## 6 CONCLUSION

The present findings demonstrated a relationship between stress, psychological symptoms and job satisfaction. The main concerns were workload, work department, supervision, collegial relationships and high mortality rates in patients. More research is needed to identify what types of support are required, along with mechanisms to tailor such support to the different variables identified by the nursing participants. Based on the findings of this study, we recommend focusing efforts on raising awareness among hospital managers regarding nurses' psychological symptoms and possible support measures, which may include flexible working hours, clear communication and training in palliative and end‐of‐life care. Finally, qualitative investigation is highly recommended to explore in‐depth further context for the identified sources of stress, and psychological and emotional experiences among nurses as frontline workers facing COVID‐19. A co‐design approach may be particularly beneficial, as this will not only lead to strategies that draw from the knowledge and experience of the nursing staff but also potentially offer these nurses the opportunity to take back some control in a time of immense instability.

## AUTHOR CONTRIBUTIONS

All authors listed have met all four of the following criteria: Have made substantial contributions to conception and design, or acquisition of data, or analysis and interpretation of data; Been involved in drafting the manuscript or revising it critically for important intellectual content; Given final approval of the version to be published. Agreed to be accountable for all aspects of the work in ensuring that questions related to the accuracy or integrity of any part of the work are appropriately investigated and resolved.

## FUNDING INFORMATION

This research received no specific grant from any funding agency in the public, commercial or not‐for‐profit sectors.

## CONFLICT OF INTEREST

The authors have no conflict of interest to declare.

## ETHICS STATEMENT

Ethical approval was obtained from the King Fahd Armed Forces Hospital—Jeddah Research and Ethics Committee (Ref. number: REC 398), confirming no risk to study participants via the application of an anonymous online survey. This study conforms to the recognized standards listed by the Declaration of Helsinki.

## Supporting information


Table S1

## Data Availability

The data that support the findings of this study are available from the corresponding author upon reasonable request.
