# Supplementary material for: Quantitative research on the impact of COVID‐19 on frontline nursing staff at a military hospital in Saudi Arabia
Source: Nurs Open. 2022 Jul 22;10(1):217–29. doi: 10.1002/nop2.1297 (PMC9349684; doi:10.1002/nop2.1297)
Supplement: Supplementary file 1 — Table S1 [file NOP2-10-217-s001.docx]

**Supplemental table 1.** **Regression coefficients of significant variables which are related DASS scores by using stepwise multiple regression analysis**

| Model | | Unstandardized coefficients | | Standardized coefficients | t-value | p-value |
| --- | --- | --- | --- | --- | --- | --- |
|  |  | B | Std. error | Beta |  |  |
| 1 | (Constant) | 29.969 | 1.194 |  | 25.091 | <0.0001 |
|  | Saudi | 18.945 | 5.043 | .149 | 3.756 | <0.0001 |
| 2 | (Constant) | 28.406 | 1.290 |  | 22.016 | <0.0001 |
|  | Saudi | 20.509 | 5.035 | .161 | 4.073 | <0.0001 |
|  | Emergency | 10.122 | 3.282 | .122 | 3.084 | .002 |
| 3 | (Constant) | 27.163 | 1.378 |  | 19.706 | <0.0001 |
|  | Saudi | 18.119 | 5.105 | .142 | 3.549 | <0.0001 |
|  | Emergency | 11.364 | 3.307 | .137 | 3.437 | .001 |
|  | Others | 8.476 | 3.406 | .101 | 2.489 | .013 |
| 4 | (Constant) | 25.088 | 1.591 |  | 15.768 | <0.0001 |
|  | Saudi | 17.725 | 5.084 | .139 | 3.486 | .001 |
|  | Emergency | 11.699 | 3.294 | .141 | 3.551 | <0.0001 |
|  | Others | 8.746 | 3.392 | .104 | 2.578 | .010 |
|  | Age 25-30 | 6.334 | 2.459 | .101 | 2.576 | .010 |

*Depression, Anxiety, and Stress scale (DASS)

**Supplemental table 2. Regression coefficients of significant variables which are related ENSS scores by using stepwise multiple regression analysis**

| Model | | Unstandardized coefficients | | Standardized coefficients | t-value | p-value |
| --- | --- | --- | --- | --- | --- | --- |
|  |  | B | Std. error | Beta |  |  |
| 1 | (Constant) | 14.181 | .653 |  | 21.721 | <0.0001 |
|  | Filipino | 4.251 | .750 | .222 | 5.669 | <0.0001 |
| 2 | (Constant) | 14.009 | .643 |  | 21.795 | <0.0001 |
|  | Filipino | 3.646 | .748 | .190 | 4.874 | <0.0001 |
|  | Emergency | 4.327 | .908 | .186 | 4.767 | <0.0001 |
| 3 | (Constant) | 13.114 | .663 |  | 19.771 | <0.0001 |
|  | Filipino | 3.944 | .740 | .206 | 5.333 | <0.0001 |
|  | Emergency | 4.944 | .904 | .212 | 5.468 | <0.0001 |
|  | Others | 4.110 | .910 | .174 | 4.519 | <0.0001 |
| 4 | (Constant) | 12.636 | .682 |  | 18.530 | <0.0001 |
|  | Filipino | 4.423 | .756 | .231 | 5.854 | <0.0001 |
|  | Emergency | 4.974 | .899 | .214 | 5.531 | <0.0001 |
|  | Others | 4.055 | .905 | .172 | 4.480 | <0.0001 |
|  | Malaysian | 6.709 | 2.420 | .107 | 2.773 | 0.006 |
| 5 | (Constant) | 11.497 | .761 |  | 15.114 | <0.0001 |
|  | Filipino | 5.619 | .834 | .293 | 6.738 | <0.0001 |
|  | Emergency | 4.996 | .892 | .215 | 5.598 | <0.0001 |
|  | Others | 3.541 | .912 | .150 | 3.884 | <0.0001 |
|  | Malaysian | 7.987 | 2.432 | .128 | 3.284 | 0.001 |
|  | Saudi | 4.976 | 1.519 | .139 | 3.275 | 0.001 |

*Extended Nursing Stress Scale (ENSS)

**Supplemental table 3. Regression coefficients of significant variables related job satisfaction scores using stepwise multiple regression analysis**

| Model | | Unstandardized coefficients | | Standardized coefficients | t-value | p-value |
| --- | --- | --- | --- | --- | --- | --- |
|  |  | B | Std. error | Beta |  |  |
| 1 | (Constant) | 117.682 | 1.112 |  | 105.822 | <0.0001 |
|  | Inpatient | 8.663 | 1.778 | .192 | 4.871 | <0.0001 |
| 2 | (Constant) | 115.642 | 1.166 |  | 99.206 | <0.0001 |
|  | Inpatient | 9.492 | 1.753 | .210 | 5.414 | <0.0001 |
|  | Indian | 12.303 | 2.470 | .193 | 4.981 | <0.0001 |
| 3 | (Constant) | 114.784 | 1.203 |  | 95.431 | <0.0001 |
|  | Inpatient | 9.992 | 1.754 | .221 | 5.695 | <0.0001 |
|  | Indian | 13.022 | 2.472 | .205 | 5.268 | <0.0001 |
|  | Saudi | 10.017 | 3.722 | .105 | 2.692 | 0.007 |
